# Endoscopic versus microscopic ear surgery in surgical education: a systematic review

**DOI:** 10.3389/fsurg.2026.1927443

**Published:** 2026-10-28

**Authors:** Lubna Khreesha, Amer Sawalha, Suhaib Abo Matar, Farah Abu Assi, Dana Al-Absi, Mohammad Hijah, Luay Abdallah, Hana Erekat

**Affiliations:** 1 Department of Special Surgery, School of Medicine, The University of Jordan & Jordan University Hospital, Amman, Jordan; 2 School of Medicine, The University of Jordan, Amman, Jordan; 3 College of Medicine, University of Sharjah, Sharjah, United Arab Emirates

**Keywords:** endoscopic ear surgery, microscopic ear surgery, otology, surgical education, training

## Abstract

**Objective:**

To systematically review the educational value of endoscopic ear surgery (EES) compared to microscopic ear surgery (MES) in otolaryngology training programs.

**Data sources:**

PubMed, Web of Science, Cochrane Library, and Scopus (up to October 2025).

**Review methods:**

A systematic review was conducted following PRISMA guidelines. Studies comparing EES and MES in educational settings (cadaveric, live surgery, or simulation) involving medical students, residents, or fellows were included. Outcomes were categorized into cognitive (anatomical knowledge), psychomotor (technical skills), and affective (perceived educational value) domains. Risk of bias was assessed using RoB 2, ROBINS-I, and JBI tools, and the certainty of evidence was evaluated using the GRADE approach.

**Results:**

Eight studies (*n* = 287 participants) met the inclusion criteria. In the affective domain, EES was consistently rated higher for perceived educational value, optical quality, and anatomical visualization, particularly by junior trainees. In the cognitive domain, objective assessments of anatomical knowledge acquisition showed no significant difference between modalities, though EES provided a shared perspective beneficial for early orientation. In the psychomotor domain, MES was frequently preferred by senior trainees for developing bimanual dexterity and depth perception. The overall certainty of evidence was low to moderate due to study heterogeneity and reliance on subjective measures.

**Conclusion:**

EES and MES offer complementary educational benefits. EES excels in early-phase anatomical orientation and shared visualization, while MES remains crucial for advanced technical skill refinement. Otolaryngology curricula should integrate both modalities sequentially to optimize surgical competency.

## 1 Introduction

Otological disorders represent a major global health burden. Hearing loss ranks as the fourth most prevalent chronic disease worldwide, and conditions such as chronic suppurative otitis media—affecting up to 10% of populations in low- and middle-income countries—cholesteatoma, and other middle ear pathologies pose substantial threats to hearing, balance, and quality of life (1–3). Because the middle ear is among the most technically demanding and anatomically constrained regions of the body, effective surgical visualization is paramount.

Microscopic ear surgery (MES) has served as the gold standard for otological procedures since its introduction in 1951, offering binocular magnification and reliable depth perception through a postauricular approach. However, MES is limited by a straight-line operative field that restricts visualization of hidden middle ear recesses and often necessitates more invasive incisions to achieve adequate exposure. These constraints have driven the evolution of endoscopic ear surgery (EES), which emerged in the 1990s and offers panoramic visualization of anatomically challenging regions with reduced postoperative pain and improved cosmetic outcomes (4–6). Despite its advantages, EES presents its own challenges: hemostasis is more demanding because the endoscope's field of view is severely compromised by even modest bleeding, and the technique carries a steeper initial learning curve, raising questions about its practical adoption in training programmes (7).

Surgical training in otolaryngology has been further complicated by declining operative case volumes in many residency programmes (8). The shift towards competency-based medical education (CBME) necessitates efficient and effective training modalities. Simulation-based approaches—including cadaveric training and, more recently, augmented and virtual reality platforms—have been proposed to fill this gap, but remain constrained by limited access to cadaveric materials and the relative immaturity of simulation models (9, 10). MES aligns naturally with binocular microscopy training, enabling relatively rapid technical mastery. In contrast, EES demands adaptation to monocular two-dimensional visualization, bimanual coordination that incorporates suction in the non-dominant hand, and an inherently single-handed instrument technique—all of which increase operative time and complexity during the learning phase (11, 12). Conversely, several investigators have argued that training on EES may be advantageous for junior surgeons because the endoscope's single shared video feed provides supervisor and trainee with an identical, unambiguous vantage point by default (13). It should be noted that MES can also be projected to a 2D or 3D monitor for the supervising surgeon, and some microscopes incorporate a co-observation eyepiece; the distinction is therefore not the availability of a shared view *per se*, but that with EES this shared view is the primary mode of visualization for the operating surgeon as well, rather than an optional add-on. Trainees with prior endoscopic experience—for instance, in functional endoscopic sinus surgery—also appear to adapt more rapidly to EES (14).

Educational value in this context encompasses not only the speed of skill acquisition but also anatomical comprehension, the transferability of acquired skills across techniques, error recognition, and patient safety. To date, no comprehensive systematic review has synthesized the available evidence comparing the educational impact of EES and MES. Understanding whether one visualization modality confers consistent advantages in anatomical comprehension, technical skill acquisition, or trainee satisfaction is essential for informing contemporary otolaryngology curricula.

This systematic review aims to address this gap by comparing the educational value of EES versus MES across cognitive, psychomotor, and affective domains, with subgroup consideration of learner experience level.

## 2 Methods

### 2.1 Study registration and reporting

This systematic review was conducted and reported in accordance with the Preferred Reporting Items for Systematic Reviews and Meta-Analyses (PRISMA) 2020 guidelines (15, 16). The review protocol was prospectively registered on PROSPERO (registration number: CRD420251166206) on 11 October 2025.

### 2.2 Search strategy

A systematic search was conducted across four electronic databases—PubMed, Web of Science, the Cochrane Library, and Scopus—for articles indexed up to 11 October 2025. The search strategy combined terms related to endoscopic and microscopic ear surgery with educational outcomes. The full search strategy for PubMed, Scopus, Web of Science, and Cochrane is provided in Appendix, advanced search has been used in the last two. Reference lists of all included studies were manually screened to identify additional eligible articles. The search term blocks used across all databases are provided in the Appendix; database-specific syntax, search modes, and retrieved records are summarized in Supplementary Table 1.

**Table 1:** Characteristics of included studies.

Study ID	Country	Study Design	Population	*N*	Training Context
Anschuetz et al. (20)	Switzerland	Randomized crossover study	Specialists, residents, and 6th-year medical students	33	Cadaveric temporal bone
Anschuetz et al. (22)	Switzerland	Randomized crossover study	Consultants, residents, and final-year medical students	33	Cadaveric temporal bone model
Anschuetz et al. (23)	Switzerland	Qualitative study (thematic analysis)	Medical students, residents, and staff members	19	Cadaveric
Chern (24)	USA	Cross-sectional observational study	Second- to fourth-year medical students	44	Live surgery (observed on screen)
Hashim et al. (25)	South Korea	Retrospective comparative study	Otolaryngology trainees	13	Live surgery
Iannella (26)	Italy	Prospective observational study	Otolaryngology residents (PGY 1–5)	21	Live stapes surgery
Tu (27)	USA	Randomized controlled educational intervention	Medical students and residents	62	Video modules (5 min each)
Niederhauser (21)	Switzerland	Randomized within-subject educational study	Medical students	62	Video modules (∼9 min each)

### 2.3 Selection criteria

Study selection was based on predefined inclusion and exclusion criteria using the PICOS framework. Two independent reviewers screened all candidate records by title and abstract, followed by full-text review of potentially eligible articles. Discrepancies were resolved by consensus. Duplicate records were excluded at the screening stage.

Studies were eligible for inclusion if they: (1) directly compared educational outcomes between EES and MES; (2) enrolled medical students, residents (PGY 1–5), or surgical fellows as participants; and (3) were conducted in educational or clinical training settings where EES and MES were used as components of surgical education. Studies focusing exclusively on patient clinical outcomes without reference to training or educational value were excluded, as were abstracts, letters, editorials, narrative reviews, and expert opinions, and any study for which a full text in English was unavailable and conference abstracts or unpublished data were not included either. The study selection process is presented in the PRISMA flow diagram (Figure 1).

**Figure 1:**
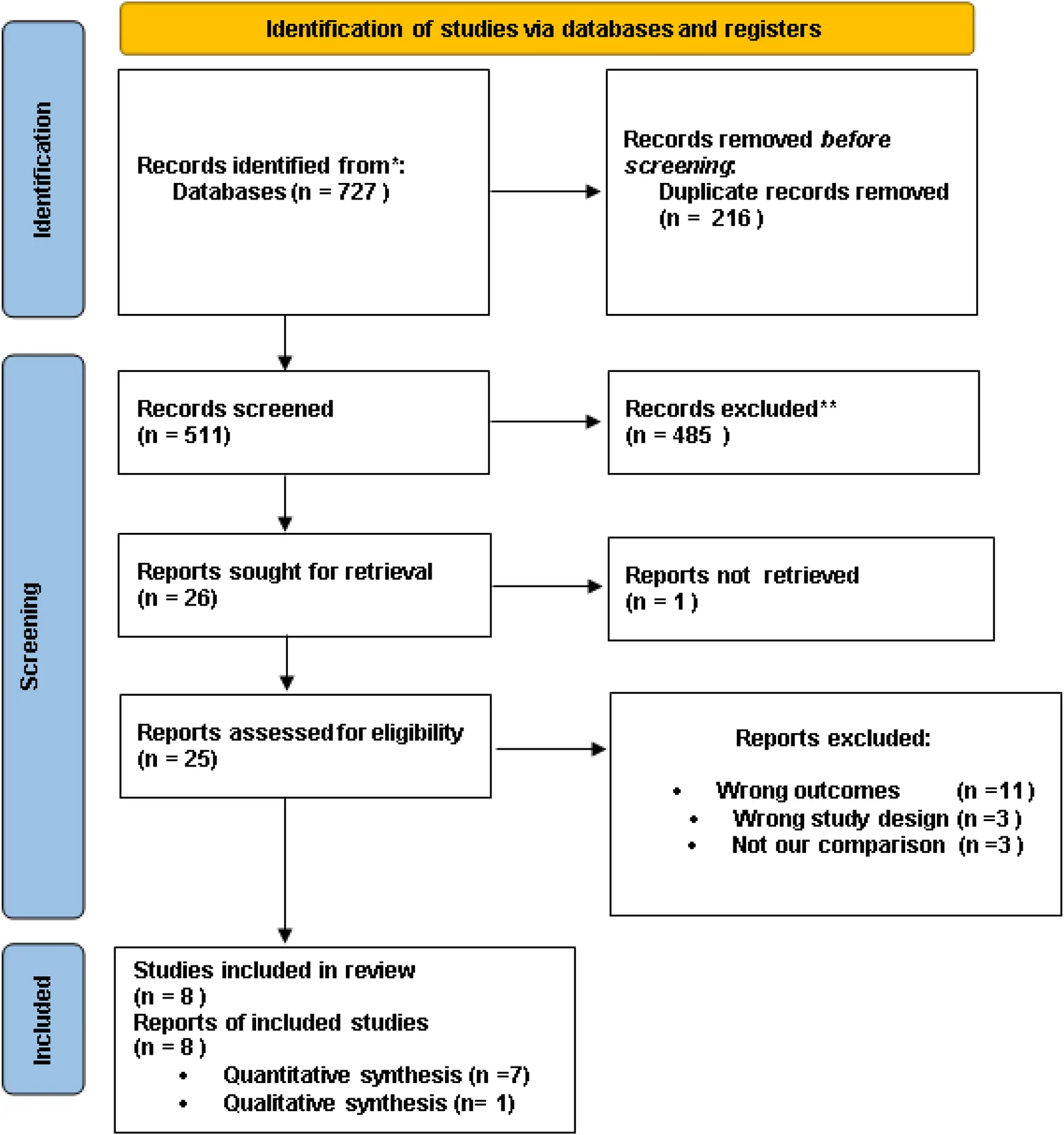
PRISMA 2020 flow diagram showing the identification, screening, eligibility assessment, and inclusion of studies included in the systematic review. Created by the authors using the PRISMA 2020 flow diagram template (Page et al., BMJ 2021;372:n71, CC BY 4.0, cited as ref. 15).

### 2.4 Data extraction

Data were extracted independently by two reviewers using a standardized data extraction form. Extracted variables included study characteristics, participant characteristics, training context, intervention and comparator details, and educational outcome measures. Outcomes were categorized into three domains: cognitive (e.g., anatomical knowledge scores), psychomotor (e.g., procedural steps, instrument handling), and affective (e.g., perceived educational value, satisfaction).

### 2.5 Risk of bias and certainty of evidence assessment

Risk of bias was assessed using study design-appropriate validated tools. The revised Cochrane Risk of Bias tool (RoB 2) was applied to randomized and quasi-randomized controlled trials (17). The Risk of Bias in Non-randomized Studies of Interventions (ROBINS-I) tool was used for non-randomized cohort studies (18). The Joanna Briggs Institute (JBI) Critical Appraisal Checklists were applied for cross-sectional and qualitative studies (19). Standard intervention risk of bias tools were not applicable for studies using qualitative focus-group designs (23) or single arm educational designs (21). Instead, methodological quality for these studies was appraised narratively. Quantitative risk of bias figures were generated with the robvis web application. The certainty of evidence for key outcomes was evaluated using the Grading of Recommendations Assessment, Development and Evaluation (GRADE) approach.

### 2.6 Data synthesis

Due to substantial clinical and methodological heterogeneity, meta-analysis was not considered appropriate. The included studies assessed different educational constructs, including anatomical knowledge, identification of specific middle ear structures, procedural performance, operative errors, visual quality, cognitive load, and learner preference. They also differed in training context, ranging from cadaveric dissection and simulation to live surgery and video-based instruction, and included participants with varying levels of experience. Outcomes were reported using non-equivalent formats, including pre- and post-intervention scores, paired crossover comparisons, independent-group ratings, Likert-scale responses, binary outcomes, and qualitative themes. Although standardized mean differences can account for variation in numerical scales, they do not resolve differences in the underlying constructs being measured, combining these outcomes would therefore have produced a pooled estimate with limited educational meaning. In addition, individual outcomes were generally reported by too few studies, or with insufficiently comparable data, to support meaningful pooling. Findings were therefore synthesized narratively within the predefined cognitive, psychomotor, and affective domains, in accordance with the Synthesis Without Meta-analysis (SWiM) reporting guideline.

## 3 Results

### 3.1 Study selection and characteristics

The search identified 727 records. After removing duplicates and screening titles/abstracts, 26 full-text articles were assessed for eligibility. Eight studies met the inclusion criteria (Table 1). The studies were published between 2017 and 2024 and originated from Switzerland (*n* = 4), the United States (*n* = 2), Italy (*n* = 1), and South Korea (*n* = 1). Study designs included randomized crossover educational studies (*n* = 2), a randomized controlled educational intervention (*n* = 1), a randomized comparative study (*n* = 1), a cross-sectional observational study (*n* = 1), a retrospective comparative study (*n* = 1), a prospective observational study (*n* = 1), and a qualitative study using thematic analysis (*n* = 1).

### 3.2 Affective domain: perceived educational value and satisfaction

The affective domain, encompassing learner perceptions, satisfaction, and perceived visual quality, was the most consistently reported outcome favoring EES. Chern et al. (25) evaluated learner perceptions during intraoperative observation and reported significantly higher ratings for EES compared with MES across multiple domains, including visibility of the operative field (4.74 vs. 4.28, *P* < 0.01), optical quality (4.78 vs. 4.28, *P* < 0.01), and overall educational value. These findings reflected improved visual access rather than objective knowledge assessment and remained consistent after adjustment for potential confounders.

Qualitative findings by Anschuetz et al. (23) highlighted improved shared visualization and anatomical orientation with EES. Junior learners reported greater benefit from shared visualization and real-time instructor guidance during endoscopic procedures. Niederhauser et al. (21) demonstrated that highest subjective ratings for image quality, depth perception, and usability were observed in the 3D-EES condition, while MES video was associated with higher cognitive load and lower learner preference.

### 3.3 Cognitive domain: anatomical knowledge acquisition

Several studies examined changes in anatomical knowledge following instructional exposure, with mixed results. Anschuetz et al. (20), using objective pre- and post-intervention testing within a structured educational curriculum, reported greater absolute gains in anatomical understanding among participants trained using endoscopic visualization, particularly among less experienced learners.

In contrast, Tu et al. (27) reported significant post-intervention improvements in anatomy knowledge in both EES and MES groups following video-based instruction, with no statistically significant difference between approaches after adjustment for baseline knowledge. Hashim et al. (25) found that EES was associated with higher mean anatomy scores (7.23 ± 1.80 vs. 6.62 ± 1.39), but the difference did not reach statistical significance (*P* = 0.358). Iannella et al. (26), focusing on stapes surgery, reported improved identification of selected middle ear structures with endoscopic visualization (7.1 ± 0.79 vs. 6.2 ± 1.40; *P* = 0.009).

### 3.4 Psychomotor domain: technical skills and procedural learning

Procedural learning outcomes were generally comparable between visualization techniques, though perceptions of skill acquisition varied by experience level. Anschuetz et al. (22) reported similar task-completion performance between endoscopic and microscopic approaches, although accidental ossicular contact occurred more frequently during microscopic tasks.

Qualitative data from Anschuetz et al. (23) revealed that while junior learners preferred EES for shared visualization, more experienced trainees and staff more frequently emphasized the value of MES for instrument handling, bimanual dexterity, and depth control. Hashim et al. (25) reported largely comparable educational outcomes between endoscopic and microscopic techniques regarding procedural steps understanding (7.45 ± 1.33 vs. 6.53 ± 1.42; *P* = 0.285).

### 3.5 Risk of bias and certainty of evidence

Risk of bias varied across studies (Figures 2–4). Among the randomized trials (Figures 2A,B) assessed using RoB 2, three demonstrated low overall risk of bias. Non-randomized cohort studies (Figure 3) evaluated using ROBINS-I were rated as having moderate-to-serious risk of bias, primarily attributable to confounding by learner experience and non-random allocation. The cross-sectional study (Figure 4) demonstrated a moderate risk of bias due to reliance on questionnaire based subjective evaluations. Narrative appraisal of the qualitative study and single-arm observational study revealed helpful descriptive and mechanistic findings. However, the absence of a direct control group limits the overall certainty of this evidence. The overall certainty of evidence, assessed via GRADE, was considered low to moderate for most outcomes due to study heterogeneity, small sample sizes, and reliance on subjective self-report measures.

**Figure 2:**
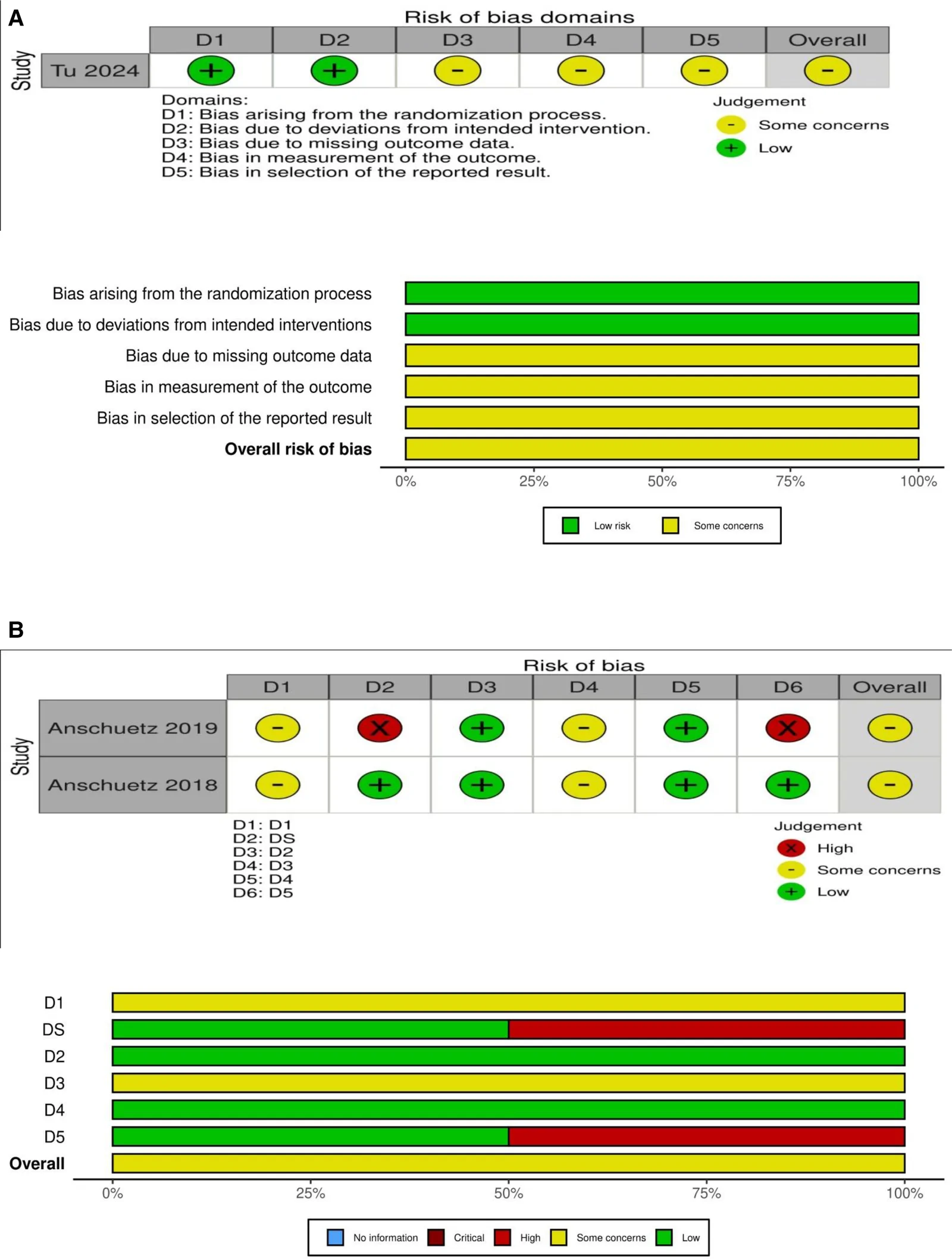
**(A)** parallel RCT assessment. **(B)** Crossover RCTs Assessment. Figure created using the robvis web application.

**Figure 3:**
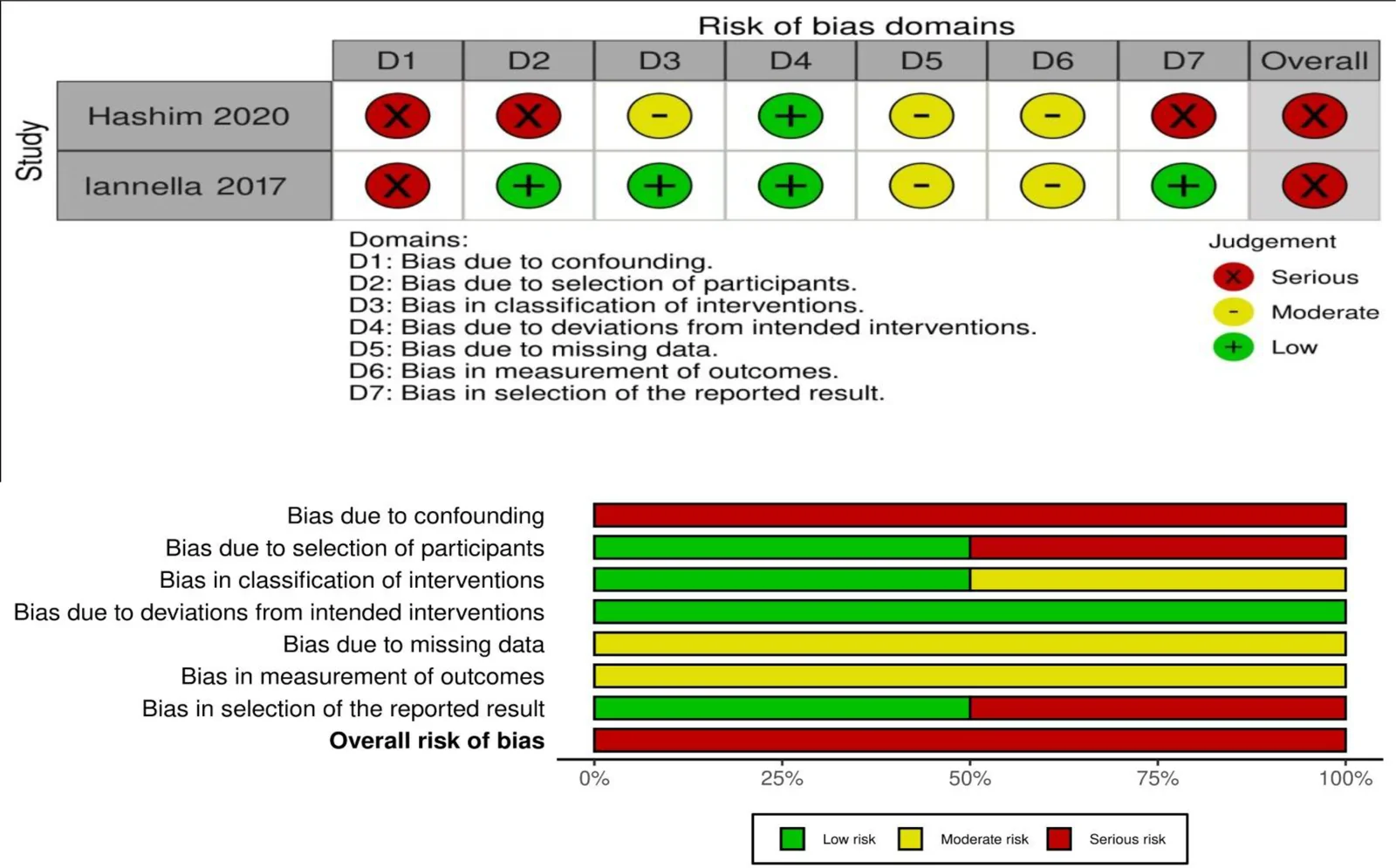
ROBINS-I assessment. Figure created using the robvis web application.

**Figure 4:**
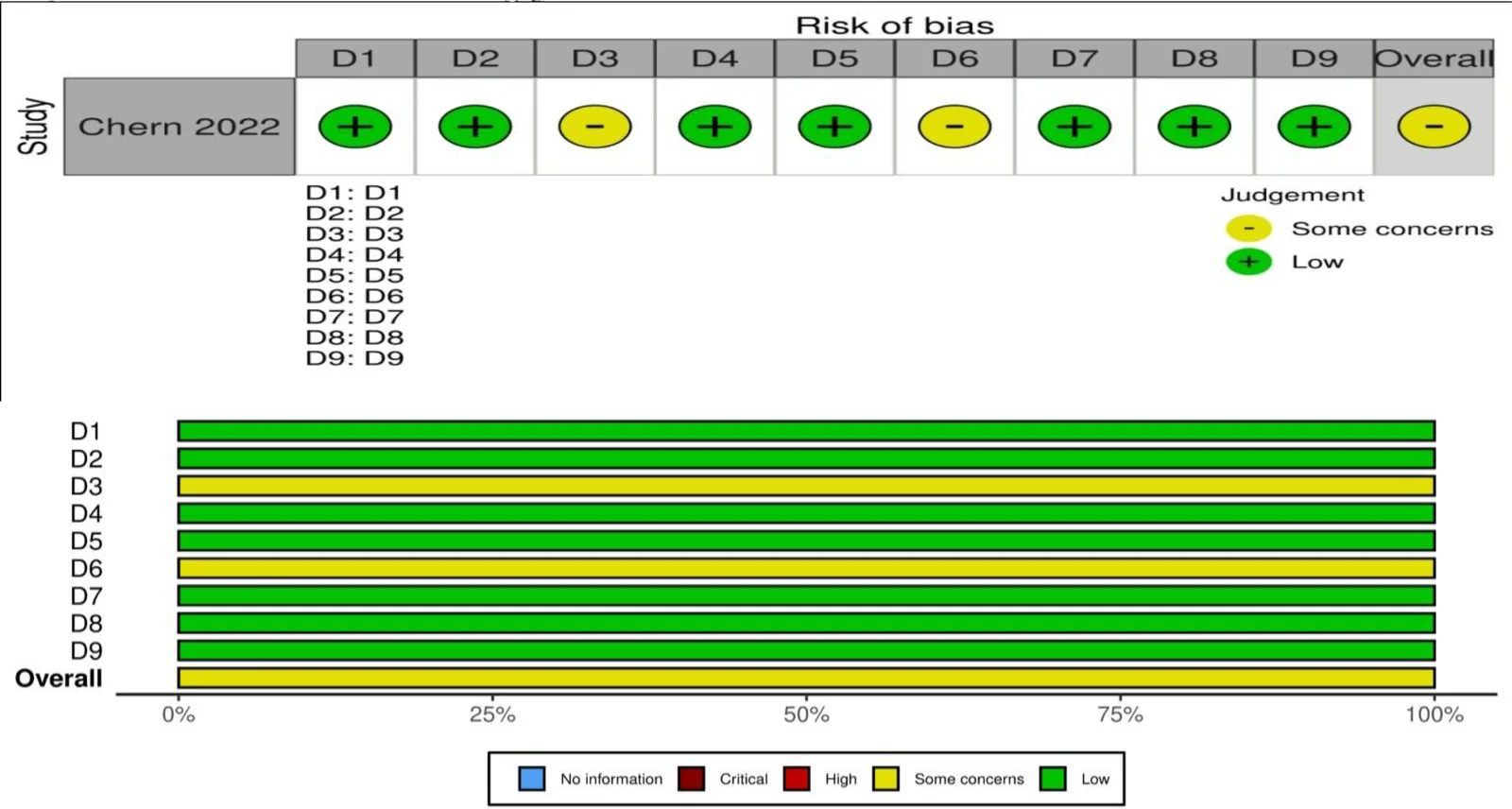
JBI critical appraisal assessment. Figure created using the robvis web application.

## 4 Discussion

This systematic review synthesizes available evidence comparing the educational value of EES and MES in surgical training. The principal finding is that neither modality demonstrates uniform superiority across all educational outcomes. Instead, they offer complementary benefits across different domains of learning and stages of training.

### 4.1 Interpretation of findings by educational domain

In the affective domain, EES is consistently favored for perceived educational value, optical quality, and anatomical visualization. The wide-angle, panoramic view afforded by the endoscope reduces the proportion of the middle ear that is obscured from the learner's perspective. While microscope-based systems can also route the operative view to a 2D or 3D monitor or an assistant eyepiece, this is often supplementary rather than the operating surgeon's own primary view; with EES, by contrast, the trainee, supervisor, and operating surgeon inherently share the same feed. This distinction, rather than a categorical advantage in visualization itself, is what several authors have proposed underlies EES's perceived educational benefit for real-time guidance (13). This shared perspective has particular relevance for surgical education, where real-time feedback and concurrent observation are central to competency development.

In the cognitive domain, objective differences in anatomy knowledge acquisition are modest. While EES provides superior visualization of hidden recesses (e.g., sinus tympani, facial recess), this does not always translate to significantly higher scores on standardized anatomy tests compared to MES. Both modalities are effective for teaching basic middle ear anatomy.

In the psychomotor domain, the educational advantages appear experience-level sensitive. More experienced trainees consistently attributed greater value to MES for the development of instrument handling, bimanual dexterity, and depth perception—domains in which the two-dimensional, single-handed nature of EES may represent a relative disadvantage.

### 4.2 Implications for surgical curricula

These findings have significant practical implications for residency program directors designing contemporary otolaryngology curricula. The data suggest a complementary rather than competitive relationship between the two modalities.

#### 4.2.1 Sequential Integration

Curricula should consider introducing EES early in training (PGY-1/2) primarily for anatomical orientation and cognitive knowledge acquisition. The shared screen allows attendings to point out structures and guide the trainee's understanding of complex 3D relationships. This anatomical rationale is further supported by cadaveric feasibility data showing that the endoscopic approach allows more complete visualization of key middle ear structures while requiring less bony scutum resection than the microscopic approach, with comparable rates of chorda tympani injury between the two techniques (28).

#### 4.2.2 Technical refinement

As trainees progress, MES should be emphasized to develop critical psychomotor skills, including bimanual dexterity, depth perception, and fine instrument handling.

#### 4.2.3 Simulation and transition

The steeper learning curve of EES, particularly regarding single-handed dissection and hemostasis, suggests a role for simulation. Trainees should achieve proficiency in endoscope handling in a cadaveric or simulated environment before transitioning to live EES cases.

#### 4.2.4 Technological advancements

The introduction of three-dimensional endoscopy may partially mitigate EES's depth perception limitations and warrants integration into training programs as the technology becomes more accessible.

#### 4.2.5 Digital and AI-assisted training tools

Beyond three-dimensional endoscopy, several emerging technologies may further shape the EES–MES educational landscape. Virtual reality temporal bone simulators allow repeated, low-stakes rehearsal of anatomical dissection without consuming cadaveric resources, and validated platforms are increasingly being incorporated into otolaryngology training curricula (29, 30). Mixed and augmented reality platforms, which overlay anatomical or navigational information onto the surgical field, have shown early and growing application in otology/neurotology, particularly for surgical navigation, with emerging but still limited evidence for their educational use (31). Separately, automated and AI-assisted skill assessment systems—using motion tracking, force sensors, and computer-vision analysis of simulator or video data—are being validated as objective, scalable alternatives to rater-dependent assessment in surgical simulation generally (32), and platform-specific tools such as automated final-product scoring for virtual reality mastoidectomy illustrate how this approach is beginning to reach otologic training specifically (33). Integration of such digital and AI-assisted competency assessment tools into EES and MES curricula could help address a key limitation of the current evidence base—reliance on subjective, self-reported outcome measures—and represents a promising direction for future educational research in this field.

### 4.3 Limitations

Several important limitations of this review must be acknowledged. First, meta-analysis was not performed because the included studies differed substantially in the outcomes assessed, the instruments used, participant experience, and training settings. Although standardized mean differences can account for variation in scoring scales, they cannot overcome differences in the underlying educational constructs, making any pooled estimate difficult to interpret. Second, sample sizes across the included studies were generally small, increasing the risk of type II error. Third, the restriction to English-language publications may introduce language bias. Fourth, most studies relied heavily on subjective self-report measures, which are susceptible to social desirability bias. Finally, none of the included studies employed long-term follow-up to assess retention of educational gains or transfer of skills to clinical practice.

Additionally, standard risk of bias tools was not applicable to all included studies. Specifically, the qualitative focus groups (23) and single-arm educational observations (21) lack independent control groups and comparative metrics. While they provide helpful learner centered and ergonomic context, their conclusions should be interpreted within their educational environment rather than as comparative clinical data.

Another limitation is our exclusive focus on educational, cognitive, and psychomotor training metrics rather than clinical patient endpoints like graft uptake or hearing outcomes. While clinical efficacy is thoroughly documented in prior literature, future research is needed to determine how trainee learning curves under 2D endoscopic versus 3D microscopic visualization directly translate into real-world operative and clinical outcomes.

## 5 Conclusion

EES and MES each offer distinct and complementary educational advantages in otological surgical training. EES excels in providing superior anatomical visualization and a shared perspective that enhances early-phase cognitive learning and trainee satisfaction. Conversely, MES remains essential for developing advanced psychomotor skills, including bimanual dexterity and depth perception. Contemporary otolaryngology curricula should integrate both modalities sequentially, leveraging EES for initial anatomical orientation and MES for technical skill refinement, to optimize surgical competency. Future research should prioritize adequately powered randomized controlled trials using objective, validated educational outcome measures with longitudinal assessment of skill retention.

## Appendix A: Search Strategy

Block A: “endoscop*” OR “transcanal endoscopic” OR “TEES” OR “EES” OR “otoendoscopy”
Block B: “microscop*” OR “operative microscopy” OR “microsurgical”
Block C: “otology” OR “ear” OR “tympano*” OR “myringo*” OR “mastoid*” OR “cholesteatoma” OR “ossiculo*” OR “staped*” OR “middle ear surgery” OR “ear surgery” OR “tympanoplasty” OR “stapedectomy”
Block D: “education” OR “training” OR “learning curve” OR “teaching” OR “skills” OR “surgical education” OR “resident education” OR “surgical simulation” OR “competency” OR “proficiency” OR “assessment”

## References

[1] Goman AM Lin FR. Prevalence of hearing loss by severity in the United States. Am J Public Health. (2016) 106(10):1820–2. 10.2105/AJPH.2016.303299 27552261 PMC5024365

[2] World Health Organization. Chronic Suppurative Otitis Media: Burden of Illness and Management Options. Geneva: WHO (2004). Available online at: https://iris.who.int/handle/10665/42941 (Accessed May 5, 2026).

[3] Castle JT. Cholesteatoma pearls: practical points and update. Head Neck Pathol. (2018) 12(3):419–29. 10.1007/s12105-018-0915-5 30069838 PMC6081285

[4] Tarabichi M. Endoscopic middle ear surgery. Ann Otol Rhinol Laryngol. (1999) 108(1):39–46. 10.1177/000348949910800106 9930539

[5] Marchioni D Soloperto D Rubini A Villari D Genovese E Artioli F. Endoscopic exclusive transcanal approach to the tympanic cavity cholesteatoma in pediatric patients: our experience. Int J Pediatr Otorhinolaryngol. (2015) 79(3):102451. 10.1016/j.amjoto.2020.102451 25631934

[6] Emre IE Cingi C Bayar Muluk N Nogueira JF. Endoscopic ear surgery. J Otol. (2020) 15(1):27–32. 10.1016/j.joto.2019.11.004 32110237 PMC7033590

[7] Barber SR Chari DA Quesnel AM. Teaching endoscopic ear surgery. Otolaryngol Clin North Am. (2021) 54(1):65–74. 10.1016/j.otc.2020.09.005 33243377

[8] Mann S Duffy J Muffly T Tilva K Gray S Hetzler L. Effect of the COVID-19 pandemic on otolaryngology trainee surgical case numbers: a multi-institutional review. Otolaryngol Head Neck Surg. (2022) 168:26–31. 10.1177/01945998221083845 PMC9880741 35290132

[9] Piromchai P Avery A Laopaiboon M Kennedy G O'Leary S. Virtual reality training for improving the skills needed for performing surgery of the ear, nose or throat. Cochrane Database Syst Rev. (2015) 9:CD010198. 10.1002/14651858.CD010198.pub2 PMC9233923 26352008

[10] Naik SM Naik MS Bains NK. Cadaveric temporal bone dissection: is it obsolete today? Int Arch Otorhinolaryngol. (2014) 18(1):63–7. 10.1055/s-0033-1351681 25992066 PMC4296941

[11] Tu LJ Fina M Golub JS Kazahaya K Quesnel AM Tawfik KO. Current trends in endoscopic ear surgery. Otol Neurotol Open. (2022) 2(4):e023. 10.1097/ONO.0000000000000023 38516579 PMC10950159

[12] Preyer S. Endoscopic ear surgery – a complement to microscopic ear surgery. HNO. (2017) 65(Suppl 1):29–34. 10.1007/s00106-016-0268-x 27933350 PMC5281654

[13] Ridge SE Shetty KR Lee DJ. Current trends and applications in endoscopy for otology and neurotology. World J Otorhinolaryngol Head Neck Surg. (2021) 7(2):101–8. 10.1016/j.wjorl.2020.09.003 33997719 PMC8103526

[14] Mijovic T Lea J. Training and education in endoscopic ear surgery. Curr Otorhinolaryngol Rep. (2015) 3:193–9. 10.1007/s40136-015-0101-1

[15] Page MJ McKenzie JE Bossuyt PM Boutron I Hoffmann TC Mulrow CD. The PRISMA 2020 statement: an updated guideline for reporting systematic reviews. Br Med J:(2021) 372:n71. 10.1136/bmj.n71 PMC8005924 33782057

[16] Page MJ Moher D Bossuyt PM Boutron I Hoffmann TC Mulrow CD. Explanation and elaboration: updated guidance and exemplars for reporting systematic reviews. Br Med J. (2020) 2021(372):n160. 10.1136/bmj.n160 PMC8005925 33781993

[17] Sterne JAC Savović J Page MJ Elbers RG Blencowe NS Boutron I. Rob 2: a revised tool for assessing risk of bias in randomised trials. Br Med J. (2019) 366:l4898. 10.1136/bmj.l4898 31462531

[18] Sterne JAC Hernán MA Reeves BC Savović J Berkman ND Viswanathan M. ROBINS-I: a tool for assessing risk of bias in non-randomised studies of interventions. Br Med J. (2016) 355:i4919. 10.1136/bmj.i4919 PMC5062054 27733354

[19] Moola S Munn Z Tufanaru C Aromataris E Sears K Sfetcu R. Chapter 7: systematic reviews of etiology and risk. In: Aromataris E Munn Z, editors. JBI Manual for Evidence Synthesis. Adelaide: JBI (2020).

[20] Anschuetz L Siggemann T Dür C Dreifuss C Caversaccio M Huwendiek S. Teaching middle ear anatomy and basic ear surgery skills: a qualitative study comparing endoscopic and microscopic techniques. Otolaryngol Head Neck Surg. (2021) 165:174–81. 10.1177/0194599820977191 33287674

[21] Niederhauser L Fink RD Mast FW Caversaccio M Anschuetz L. Video learning of surgical procedures: a randomized comparison of microscopic, 2- and 3-dimensional endoscopic ear surgery techniques. Otol Neurotol. (2022) 43(7):e746–52. 10.1097/MAO.0000000000003550 35763494

[22] Anschuetz L Huwendiek S Stricker D Yacoub A Wimmer W Caversaccio M. Assessment of middle ear anatomy teaching methodologies using microscopy versus endoscopy: a randomized comparative study. Anat Sci Educ. (2019) 12(5):507–17. 10.1002/ase.1837 30430760

[23] Anschuetz L Stricker D Yacoub A Wimmer W Caversaccio M Huwendiek S. Acquisition of basic ear surgery skills: a randomized comparison between endoscopic and microscopic techniques. BMC Med Educ. (2019) 19(1):357. 10.1186/s12909-019-1803-8 31521153 PMC6744647

[24] Chern A Sharma RK Maurrasse SE Drusin MA Ciarleglio AJ Golub JS. Educational value of endoscopic versus microscopic ear surgery. Ann Otol Rhinol Laryngol. (2022) 131:147–53. 10.1177/00034894211012600 33957787

[25] Hashim ND Lee SA Jang SH Moon IS. A comparison of endoscopic and microscopic inlay butterfly cartilage tympanoplasties and their educational utility. PLoS One. (2020) 15(10):e0241152. 10.1371/journal.pone.0241152 33125420 PMC7598459

[26] Iannella G Marcotullio D Re M Manno A Pasquariello B Angeletti D. Endoscopic vs microscopic approach in stapes surgery: advantages in the middle ear structures visualization and trainee’s point of view. J Int Adv Otol. (2017) 13(1):14–20. 10.5152/iao.2017.3322 28555595

[27] Tu LJ Nwosu OI Chieffe D Daniel E Cohen MS. Endoscopic and microscopic video modules are effective for teaching middle ear anatomy. Laryngoscope Investig Otolaryngol. (2024) 9(6):e70044. 10.1002/lio2.70044 39664780 PMC11632898

[28] Blijleven EE Willemsen K Bleys RLAW Stokroos RJ Wegner I Thomeer HGXM. Endoscopic vs. Microscopic stapes surgery: an anatomical feasibility study. Front. Surg. (2022) 9:1054342. 10.3389/fsurg.2022.1054342 36504579 PMC9727136

[29] Zagury-Orly I Solinski MA Nguyen LH Young M Drozdowski V Bain PA. What is the current state of extended reality use in otolaryngology training? A scoping review. Laryngoscope. (2023) 133(2):227–34. 10.1002/lary.30174 35548939

[30] Bolton L Young K Ray J Chawdhary G. Virtual temporal bone simulators and their use in surgical training: a narrative review. J Laryngol Otol. (2024) 138(4):356–60. 10.1017/S0022215123002025 37973532

[31] Torborg SR Kligerman MP Cohen M Schefflein J. Extended reality applications in otolaryngology beyond the operating room: a scoping review. J Clin Med. (2024) 13(21):6295. 10.3390/jcm13216295 39518434 PMC11546070

[32] Khalifa A Tahhan O Albazooni M Saeed M Hamdi R Stanners M. Automated and artificial intelligence (AI)-derived performance assessment in surgical simulation: a systematic review. Cureus. (2025) 17(12):e100477. 10.7759/cureus.100477 41552167 PMC12807625

[33] Mikkelsen PT Sørensen MS Senn P Frithioff A Andersen SAW. Automatic final-product assessment of virtual reality mastoidectomy performance: a validity and reliability study. Otol Neurotol. (2025) 46(1):96–103. 10.1097/MAO.0000000000004346 39511745

