# Supplementary material for: Endoscopic versus microscopic ear surgery in surgical education: a systematic review
Source: Front Surg. 2026 Oct 28;13:1927443. doi: 10.3389/fsurg.2026.1927443 (PMC13752812; doi:10.3389/fsurg.2026.1927443)
Supplement: Supplementary file 1 [file table1.docx]

**Supplementary Table 1**

*Summary of Database Search Syntax, Modes, and Retrieved Records*

| **Database** | **Search Mode &Field Tags** | **Combined Query String** | **Records (N)** |
| --- | --- | --- | --- |
| PubMed | Title/Abstract ([Title/ Abstract]) | Block A AND Block B AND Block C AND Block D | 178 |
| Scopus | Title, Abstract, Keywords (TITLE-ABS-KEY) | Block A AND Block B AND Block C AND Block D | 307 |
| Web of Science | Advanced Search – Topic (TS=) | Block A AND Block B AND Block C AND Block D | 211 |
| Cochrane Library | Advanced Search – Keyword  (:ti,ab,kw) | Block A AND Block B AND Block C AND Block D | 31 |
| Total records identified 727  Total after duplicates removed 511 | | | |
